# The relationship between Oxidation Balance Score and all-cause mortality in patients with hypertension: An observational study

**DOI:** 10.1097/MD.0000000000046060

**Published:** 2025-11-21

**Authors:** Jie Li, Yiming Zeng, Yue Guan, Chuang Sun, Mengmeng Shan, Jinzhou Zhu, Qiying Yao, Liang Chen

**Affiliations:** a Department of Cardiology, Ruijin-Hainan Hospital Shanghai Jiao Tong University School of Medicine (Hainan Boao Research Hospital), Qionghai, China; b Department of Cardiology, The Second Affiliated Hospital of Dalian Medical University, Dalian, China; c Basic Medicine College, Dalian Medical University, Dalian, China.

**Keywords:** all-cause mortality, hypertension, NHANES, Oxidation Balance Score

## Abstract

The Oxidation Balance Score (OBS), which integrates prooxidant and antioxidant exposures from diet and lifestyle, serves as an indicator of systemic oxidative stress. This study examined the association between OBS and all-cause mortality among U.S. adults with hypertension. OBS was derived from 16 dietary nutrients and 3 lifestyle factors. The nonlinear relationship between OBS and mortality was assessed using smoothing splines. Kaplan–Meier curves, multivariable Cox regression, competing risk models, and subgroup analyses were employed to evaluate mortality risks. The results from the smoothing spline plots and Kaplan–Meier curves suggested that higher OBS levels were associated with lower all-cause mortality. After full adjustment, the Cox regression model revealed that higher OBS levels were associated with a 13% reduction in the risk of all-cause mortality among patients with hypertension compared to those with lower OBS levels (hazard ratios = 0.87, 95% confidence interval: 0.78–0.96, *P* = .004; *P* for trend = .003). Sensitivity analysis confirmed a protective association between higher OBS and 10-year mortality, indicating a 13% reduction in risk (hazard ratios = 0.87, 95% confidence interval: 0.78–0.98, *P* = .019; *P* for trend = .017). Competing risk models further confirmed that higher OBS reduced hypertension-related mortality. A nonlinear relationship exists between OBS and all-cause mortality in patients with hypertension. Higher OBS is linked to lower mortality, suggesting its potential utility in the prognostic risk assessment for hypertension.

## 1. Introduction

Hypertension is one of the leading causes of death and disability worldwide.^[1]^ Between 1990 and 2019, the number of people with hypertension increased from 650 million to 1.3 billion.^[2]^ Hypertension is a major risk factor for cardiovascular disease (CVD).^[3]^ Elevated blood pressure is associated with various cardiovascular and kidney diseases, it is the most modifiable risk factor for CVD-related disability.^[4,5]^ Under normal physiological conditions, reactive oxygen species (ROS) control vascular functions through redox signaling and vascular tone maintenance. However, under pathological conditions, excessive production of ROS (i.e., oxidative stress) leads to vascular damage and elevated blood pressure.^[6]^ Oxidative stress, inflammation, and vascular aging are molecular drivers and key mechanisms of endothelial dysfunction and arterial damage, which are major triggers^[7]^ for hypertension and cardiovascular events.

Diet and lifestyle are key modifiable factors influencing blood pressure homeostasis and oxidative balance.^[8,9]^ Disruption of this balance leads to oxidative stress and chronic inflammation.^[10]^ The Oxidation Balance Score (OBS), which integrates dietary and lifestyle-derived prooxidants and antioxidants, serves as a comprehensive marker of systemic oxidative stress.^[11]^ Studies have shown that higher OBS is negatively associated with metabolic syndrome, diabetes, CKD, hyperuricemia, and CVD,^[12–14]^ highlighting its value in assessing chronic disease prognosis.

While previous studies have linked OBS to reduced all-cause mortality in diabetes, its prognostic role specifically in hypertension remains unexplored. Given the central role of oxidative stress in hypertension pathogenesis and progression, we hypothesize that OBS may serve as a novel, integrative marker for risk stratification and personalized management in this population. This study investigates the association between OBS and mortality risk among hypertensive individuals using National Health and Nutrition Examination Survey (NHANES) data, aiming to establish OBS as a novel tool for assessing oxidative stress and guiding personalized interventions in hypertension management.

## 2. Information and methods

### 2.1. General information

#### 2.1.1. Study population

**Figure 1.**
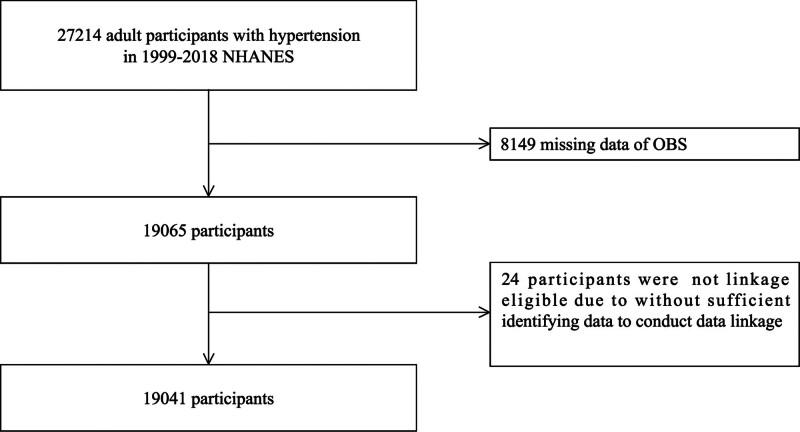
Flowchart for study population inclusion and exclusion.

The NHANES is a nationwide survey conducted by the National Center for Health Statistics. The NHANES included a total of 27,214 adult patients with hypertension from 1999 to 2018. The exclusion criterion was the absence of necessary information required to compute the OBS or assess the study outcome. Of these, 8149 participants were excluded because of missing information necessary for calculating the oxidation balance score. Additionally, 24 participants were excluded because they lacked sufficient information for the National Death Index data, resulting in missing follow-up results for survival status. A total of 19,041 participants were included in this study (Fig. 1).

#### 2.1.2. Ethical statement

The study protocol received approval from the Ethics Review Committee of the National Center for Health Statistics in the United States, and all participant provided informed consent. All experiments involved in this paper are performed in accordance with relevant guidelines and regulations.

#### 2.1.3. Definition of hypertension

Professionally trained staff measured participants’ blood pressure using standardized procedures and recorded the systolic blood pressure (SBP) and diastolic blood pressure (DBP). A questionnaire was used to inquire whether the participants were taking antihypertensive drugs. According to the guidelines of the American College of Cardiology in 2017, participants were defined as patients with hypertension if their blood pressure was greater than or equal to 130/80 mm Hg or if they were taking antihypertensive drugs.^[15]^ In the sensitivity analyses, participants were redefined as patients with hypertension if their blood pressure was ≥140/90 mm Hg or if they were taking antihypertensive drugs, according to recommendations made by the European Society of Cardiology.^[16]^

### 2.2. Observation indicators

#### 2.2.1. Oxidation Balance Score

The OBS is an indicator used to assess an individual’s oxidative stress level by evaluating the frequency of daily exposure to antioxidant and prooxidant components, including dietary and lifestyle components. Currently, there is no standardized scoring system for OBS. This study reviewed previous research and included 16 dietary nutrients and 3 lifestyle factors as scoring components. The dietary nutrients include dietary fiber, β-carotene, riboflavin, niacin, vitamin B6, total folate, vitamin B12, vitamin C, vitamin E, calcium, magnesium, zinc, copper, selenium, total fat, and iron, with total fat and iron being prooxidant components, whereas the remaining nutrients are antioxidant components. The lifestyle factors are prooxidant components, including body mass index (BMI), smoking, and alcohol consumption. Participants’ dietary data were collected through dietary recall interviews. Smoking frequency was assessed based on serum cotinine levels. Each component was assigned a score based on its tertile distribution or established cutoffs, with higher scores indicating stronger antioxidant or lower prooxidant status. The details of the scoring methods and standards are found in Table S1, Supplemental Digital Content, https://links.lww.com/MD/Q750.

#### 2.2.2. Survival status and follow-up time

The National Center for Health Statistics links mortality records from the National Death Index with the NHANES data, which includes participants’ survival status and follow-up time. All data are available on the official website (https://wwwn.cdc.gov/nchs/nhanes/Default.aspx).

#### 2.2.3. Baseline data

Patients’ baseline data were obtained through computer-assisted personal interviews, questionnaires, and blood tests. These included sex, age, race, BMI, education level, the ratio of family income to poverty, serum alanine aminotransferase (ALT), serum aspartate aminotransferase (AST), creatinine (Cr) concentration, uric acid (UA) concentration, hypercholesterolemia, diabetes, heart failure, coronary artery disease, and stroke. The definitions of hypercholesterolemia, diabetes, heart failure, and stroke were based on whether participants were previously informed by a doctor that they had these conditions. Participants were defined as having coronary artery disease if they answered “yes” to any of the following 3 questions: “Have you ever been told by a doctor that you have coronary heart disease/angina/myocardial infarction?” Missing values for ALT, AST, Cr, and UA were replaced with the mean values (missing values were <1% of the sample size).

### 2.3. Statistical methods

The weight of the sample was considered in this study. Smooth curves based on restricted cubic spline functions and Kaplan–Meier curves were used to explore the association between OBS and all-cause mortality in patients with hypertension. Regression models were used to calculate the impact of OBS on all-cause mortality risk. Stratified analyses and interaction tests were conducted to evaluate whether the results differed in the presence of different comorbidities. Sensitivity analyses were performed to verify the stability of the results. A competing risk model was also used to verify the consistency of the correlation between OBS and hypertension-related mortality. Before Cox regression and logistic regression were conducted, collinearity screening was performed on the covariates, and the results indicated that the variance inflation factor for all covariates did not exceed 5. There was no significant correlation between partial residual for OBS and rank of time, and the proportional hazards assumption is passed. Smooth curves and linear regression were used to analyze the relationship between OBS and blood pressure levels (including SBP and DBP) to explore the mechanism by which OBS mediates the prognosis of patients with hypertension.

EmpowerXYS 5.2 and Stata SE 18.5 were used for data analysis, with *P* < .05 indicating statistical significance.

## 3. Results

### 3.1. Baseline data of the study population

This study included a total of 19,041 adult patients with hypertension. After an average follow-up period of 99.49 months, 15,169 patients survived, whereas 3872 patients died. Compared with surviving patients, those who died were older and had higher levels of SBP, AST, Cr, and UA, as well as lower levels of DBP, ALT, and OBS. There were also differences in sex, race, education level, the ratio of family income to poverty, hypercholesterolemia, diabetes, heart failure, CVD, and stroke (Table 1).

**Table 1:** Baseline data of participants.

Number	All	Survival	All-cause mortality	*P*
	19,041	15,169	3872	
SBP (mm Hg)	136.41 ± 18.67	134.88 ± 17.36	142.49 ± 22.13	<.001
DBP (mm Hg)	74.07 ± 15.09	75.59 ± 14.13	68.04 ± 17.13	<.001
Time (mo)	99.49 ± 62.48	102.89 ± 63.42	86.17 ± 56.74	<.001
Age (yr)	57.80 ± 15.98	54.65 ± 15.52	70.14 ± 10.99	<.001
Sex				<.001
Male	9955 (52.28)	7824 (51.58)	2131 (55.04)	
Female	9086 (47.72)	7345 (48.42)	1741 (44.96)	
Race				<.001
Mexican American	2848 (14.96)	2325 (15.33)	523 (13.51)	
Other Hispanic	1546 (8.12)	1360 (8.97)	186 (4.80)	
Non-Hispanic White	8493 (44.60)	6213 (40.96)	2280 (58.88)	
Non-Hispanic Black	4575 (24.03)	3819 (25.18)	756 (19.52)	
Other Race	1579 (8.29)	1452 (9.57)	127 (3.28)	
Education level				<.001
<High school	5507 (28.92)	3948 (26.03)	1559 (40.26)	
High school graduate or general equivalency diploma	4651 (24.43)	3702 (24.41)	949 (24.51)	
>High school	8858 (46.52)	7504 (49.47)	1354 (34.97)	
Unknown	25 (0.13)	15 (0.10)	10 (0.26)	
Ratio of family income to poverty				<.001
<1	3371 (17.70)	2630 (17.34)	741 (19.14)	
1–3	7680 (40.33)	5804 (38.26)	1876 (48.45)	
≥3	6244 (32.79)	5331 (35.14)	913 (23.58)	
Unknown	1746 (9.17)	1404 (9.26)	342 (8.83)	
Hypercholesterolemia				<.001
No	10,759 (56.50)	8683 (57.24)	2076 (53.62)	
Yes	8282 (43.50)	6486 (42.76)	1796 (46.38)	
Diabetes				<.001
No	15,394 (80.85)	12,548 (82.72)	2846 (73.50)	
Yes	3647 (19.15)	2621 (17.28)	1026 (26.50)	
Heart failure				<.001
No	18,041 (94.75)	14,648 (96.57)	3393 (87.63)	
Yes	1000 (5.25)	521 (3.43)	479 (12.37)	
Cardiovascular disease				<.001
No	16,811 (88.29)	13,852 (91.32)	2959 (76.42)	
Yes	2230 (11.71)	1317 (8.68)	913 (23.58)	
Stroke				<.001
No	17,918 (94.10)	14,513 (95.68)	3405 (87.94)	
Yes	1123 (5.90)	656 (4.32)	467 (12.06)	
ALT (U/L)	37.25 ± 30.42	39.25 ± 29.30	29.39 ± 33.32	<.001
AST (U/L)	26.24 ± 17.90	26.14 ± 17.92	26.63 ± 17.81	.135
Creatinine (µmol/L)	84.75 ± 53.07	81.00 ± 40.75	99.44 ± 84.12	<.001
Uric acid (µmol/L)	343.27 ± 88.14	340.07 ± 85.75	355.81 ± 95.92	<.001
OBS	19.72 ± 6.98	19.98 ± 6.99	18.70 ± 6.85	<.001
OBS quartile				<.001
Q1 (4–13)	4429 (23.26)	3348 (22.07)	1081 (27.92)	
Q2 (14–19)	4803 (25.22)	3753 (24.74)	1050 (27.12)	
Q3 (20–24)	4261 (22.38)	3446 (22.72)	815 (21.05)	
Q4 (25–35)	5548 (29.14)	4622 (30.47)	926 (23.92)	

ALT = alanine aminotransferase, AST = aspartate aminotransferase, DBP = diastolic blood pressure, OBS = oxidative balance score, SBP = systolic blood pressure.

### 3.2. Analysis of the association between OBS and all-cause mortality in patients with hypertension

**Figure 2.**
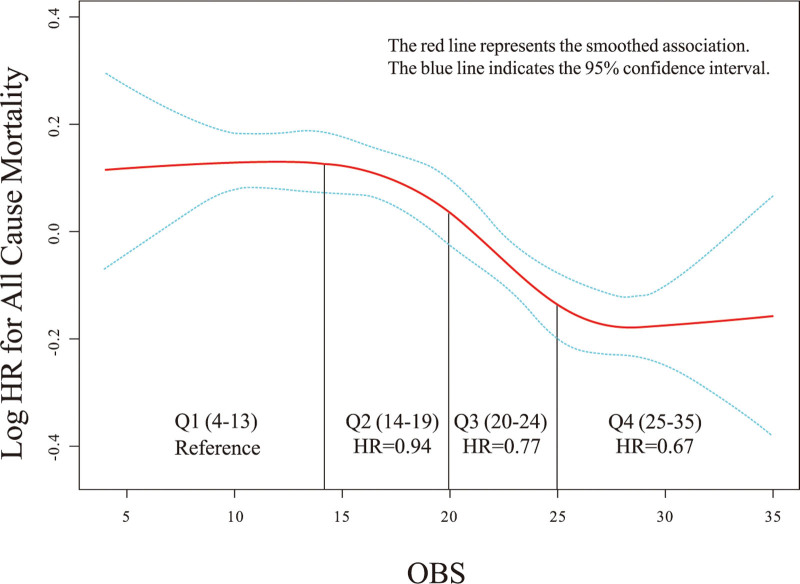
Smooth curve fitting between OBS and all-cause mortality in patients with hypertension. HR = hazard ratios; OBS = oxidative Balance Score.

**Figure 3.**
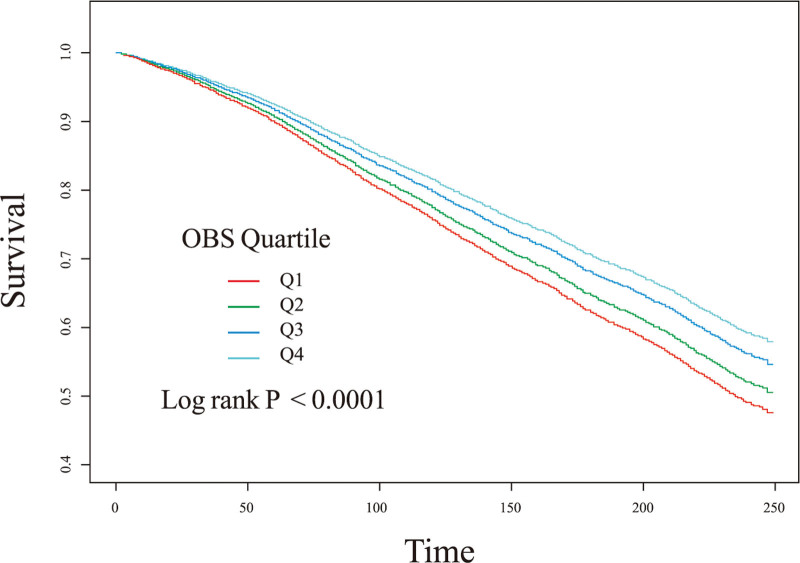
Kaplan–Meier curve of OBS quartile levels and 10-year survival rates in patients with hypertension. CI = confidence interval; HR = hazard ratios; OBS = Oxidative Balance Score.

Smooth curves indicated that as the OBS increased, the risk of all-cause mortality in patients with hypertension gradually decreased (Fig. 2). The Kaplan–Meier curve also suggested that a higher OBS was associated with a greater survival rate among patients with hypertension (Fig. 3). Table 2 shows the results of the Cox regression analysis between OBS and all-cause mortality in patients with hypertension. After adjusting for potential confounders, OBS remained significantly associated with a reduced risk of all-cause mortality in hypertensive patients. Each one-point increase in OBS was associated with a 1% decrease in all cause mortality risk (hazard ratios = 0.99, 95% confidence interval [CI]: 0.98–0.99, *P* < .001). Furthermore, when analyzed by quartiles, participants in the highest OBS group had a 21% lower risk of death compared to the lowest group (hazard ratios = 0.79, 95% CI: 0.68–0.91, *P* = .001, *P* for trend < .001). The effect estimates per standard deviation increase in OBS on all-cause mortality can be found in Table S3, Supplemental Digital Content, https://links.lww.com/MD/Q750. Additionally, the smooth curve demonstrated that as OBS increased, SBP gradually decreased (Fig S1, Supplemental Digital Content, https://links.lww.com/MD/Q750), and DBP levels showed a fluctuating decline (Fig. S2, Supplemental Digital Content, https://links.lww.com/MD/Q750). Compared to those with a lower OBS, hypertensive patients with a higher OBS exhibited significantly lower SBP by 1.15 mm Hg (β = −1.15, 95% CI: −1.93 to −0.38, *P* = .004, *P* for trend = .002) (Table S2, Supplemental Digital Content, https://links.lww.com/MD/Q750).

**Table 2:** Cox regression between OBS and all-cause mortality in patients with hypertension (weighted).

	Model 1	Model 2	Model 3
	HR (95% CI) *P*	HR (95% CI) *P*	HR (95% CI) *P*
OBS continuous	0.98 (0.97–0.98) <.001	0.97 (0.96–0.98) <.001	0.99 (0.98–0.99) <.001
OBS quartile			
Q1	Reference	Reference	Reference
Q2	0.94 (0.82–1.08) .379	0.84 (0.75–0.94) .002	0.96 (0.84–1.09) .385
Q3	0.77 (0.69–0.86) <.001	0.74 (0.66–0.83) <.001	0.88 (0.77–1.00) .050
Q4	0.67 (0.59–0.77) <0.001	0.62 (0.55–0.70) <.001	0.79 (0.68–0.91) .001
*P* for trend	<.001	<.001	<.001

Model 1: No covariates adjusted.

Model 2: Adjusted age, sex, and race.

Model 3: Adjusted age, sex, race, education level, ratio of family income to poverty, ALT, creatinine, uric acid, hyperlipidemia, diabetes, heart failure, coronary heart disease, and stroke.

ALT = alanine aminotransferase, CI = confidence interval, HR = hazard ratios, OBS = Oxidative Balance Score.

### 3.3. Stratified analysis and interaction test analysis

**Figure 4.**
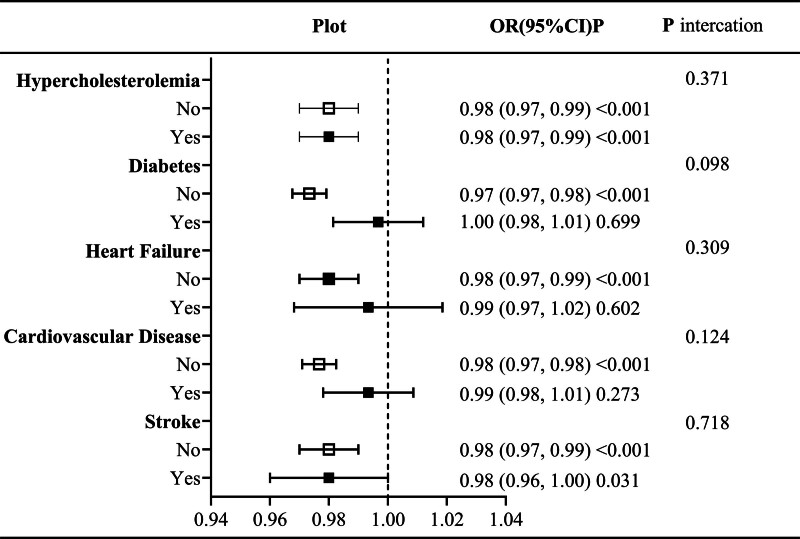
Subgroup analysis and interaction testing. Adjusted age, sex, race, education level, ratio of family income to poverty, ALT, creatinine, uric acid, hyperlipidemia, diabetes, heart failure, coronary heart disease, and stroke (excluding stratification factors). ALT = alanine aminotransferase; CI = confidence interval; OR = odds ratio.

Stratified analysis and interaction tests were conducted to confirm whether the results differed with the presence of different comorbidities. The results suggested that no effect modifiers were found (Fig. 4).

### 3.4. Sensitivity analysis

In the sensitivity analysis, hypertension was redefined according to the European Society of Cardiology guidelines as blood pressure ≥ 140/90 mm Hg or if participants were taking antihypertensive medication. A total of 13,778 patients with hypertension were included in the sensitivity analysis. In addition, 10-year all cause mortality is used as a new outcome to evaluate the stability of the results. Compared with previous results, there were no significant changes (Table 3).

**Table 3:** Sensitivity analysis (weighted).

	Model 1	Model 2	Model 3
	HR (95% CI) *P*	HR (95% CI) *P*	HR (95% CI) *P*
**Using 140/90 mm Hg to define hypertension (n = 13, 778**)			
OBS continuous	0.98 (0.97–0.98) <.001	0.97 (0.97–0.98) <.001	0.99 (0.98–0.99) <.001
OBS quartile			
Q1	Reference	Reference	Reference
Q2	0.90 (0.79–1.03) .119	0.84 (0.75–0.94) .003	0.95 (0.84–1.08) .428
Q3	0.73 (0.64–0.83) <.001	0.72 (0.64–0.82) <.001	0.85 (0.74–0.98) .028
Q4	0.66 (0.57–0.75) <.001	0.61 (0.54–0.70) <.001	0.78 (0.68–0.90) <.001
*P* for trend	<.001	<.001	<.001
**Using 10-year all cause mortality to define outcome (n = 19, 041**)			
OBS continuous	0.98 (0.97–0.98) <.001	0.97 (0.96–0.98) <.001	0.99 (0.98–0.99) .002
OBS quartile			
Q1	Reference	Reference	Reference
Q2	0.89 (0.77–1.03) .106	0.80 (0.70–0.91) <.001	0.93 (0.80–1.08) .355
Q3	0.72 (0.63–0.82) <.001	0.70 (0.61–0.81) <.001	0.85 (0.73–0.99) .043
Q4	0.64 (0.54–0.74) <.001	0.60 (0.52–0.69) <.001	0.79 (0.68–0.92) .003
*P* for trend	<.001	<.001	.002

Model 1: No covariates adjusted.

Model 2: Adjusted age, sex, and race.

Model 3: Adjusted age, sex, race, education level, ratio of family income to poverty, ALT, creatinine, uric acid, hyperlipidemia, diabetes, heart failure, coronary heart disease, and stroke.

ALT = alanine aminotransferase, CI = confidence interval, HR = hazard ratios, OBS = Oxidative Balance Score.

### 3.5. Competing risk model

A competing risk model was applied to investigate the impact of OBS on hypertension-related mortality. The results indicated that higher OBS significantly reduced the risk of hypertension-related mortality. Specifically, each one-point increase in OBS was associated with a 1% reduction in the risk of hypertension-related mortality (subhazard ratio = 0.99, 95% CI: 0.98–0.99, *P* = .047). Furthermore, when analyzed by quartiles, participants in the highest OBS group had a 25% lower risk of death compared to the lowest group (subhazard ratio = 0.75, 95% CI: 0.59–0.95, *P* = .017, *P* for trend = .049) (Table 4).

**Table 4:** Competing risk model of OBS and hypertension-related mortality.

	Death of hypertension	All other cause death
	SHR (95% CI) *P*	SHR (95% CI) *P*
OBS continuous	0.99 (0.98–0.99) .047	0.99 (0.98–0.99) .004
OBS quartile		
Q1	Ref.	Ref.
Q2	0.76 (0.61–0.94) .010	0.99 (0.90–1.10) .924
Q3	0.86 (0.69–1.08) .204	0.93 (0.83–1.03) .171
Q4	0.75 (0.59–0.95) .017	0.88 (0.79–0.98) .025
*P* for trend	.049	.011

Adjusted age, sex, race, education level, ratio of family income to poverty, ALT, creatinine, uric acid, hyperlipidemia, diabetes, heart failure, coronary heart disease, and stroke.

ALT = alanine aminotransferase, CI = confidence interval, OBS = oxidative balance score, SHR = sub-distribution risk ratio.

## 4. Discussion

This is the first study to explore the relationship between OBS and long-term survival in patients with hypertension within a large, prospective cohort. We used OBS to assess the impact of diet and lifestyle on oxidative stress and found a negative nonlinear relationship between OBS and all-cause mortality in patients with hypertension. Higher OBS was associated with reduced all-cause mortality in these patients. This negative association persisted even after confounders were adjusted. The competing risk model confirmed that higher OBS significantly reduced the risk of hypertension-related mortality. Additionally, the study showed that a high OBS was associated with reduced SBP. This study highlights the importance of adhering to an antioxidant-rich diet and healthy lifestyle to improve the prognosis of patients with hypertension, providing guidance and evidence for dietary and lifestyle modifications in patient population.

Oxidative stress, characterized by an imbalance between prooxidants and antioxidants, plays a pivotal role in the pathogenesis and progression of hypertension through 2 primary mechanisms: direct damage by reactive species and dysregulated redox signaling.^[17]^ Excessive oxidative stress is a pathogenic mediator of hypertension and a molecular trigger for CVD.^[10]^ The renin–angiotensin–aldosterone system, particularly angiotensin II, activates NADPH oxidase (NOX), leading to superoxide production. This superoxide quenches nitric oxide, promotes peroxynitrite formation, reduces nitric oxide bioavailability, and induces vasoconstriction and subsequent hypertension.^[18,19]^ CVD often develops in patients exposed to hypertension for a long time, may be attributed to target organs increased resistance or because mild blood pressure elevation has existed for a long time.^[20]^ Therefore, assessing the oxidative balance status in patients with hypertension is crucial for controlling blood pressure levels, reducing the risk of CVD, and improving prognosis.

The OBS integrates dietary and lifestyle-derived antioxidants and prooxidants, with higher scores indicating stronger antioxidant capacity. Initially applied in mortality risk assessment among smokers, OBS has since been expanded to include nutrients such as vitamin E and folate, as well as lifestyle factors like smoking and alcohol consumption.^[21–23]^ Evidence links higher OBS to longer leukocyte telomere length and reduced risk and improved prognosis of various chronic diseases, including hypertension.^[24–29]^ Consistent with previous studies, our findings show that higher OBS is negatively associated with poor long-term outcomes in hypertensive patients and is also correlated with lower SBP. Poor blood pressure control, especially SBP, is associated with adverse outcomes in patients with hypertension.^[30–32]^ The elevated SBP is a major risk factor for cardiovascular events and mortality,^[33]^ and that intensive SBP control significantly reduces cardiovascular risk.^[34]^ Our study suggests that maintaining a high OBS level may contribute to better blood pressure management and reduced all-cause mortality in hypertension.

Diets rich in fruits, vegetables, whole grains, dairy products, and plant-based proteins and diets low in red meat, processed meats, sodium, and added sugars are associated with healthy cellular aging, with particular importance placed on increasing antioxidant intake.^[35,36]^ Many antioxidants are obtained through diet,^[37]^ such as vitamin C, E, selenium, and carotenoids, which are associated with reduced CVD, cancer, and all-cause mortality.^[38]^ These antioxidants can limit the damaging effects of free radicals, especially vitamin E negatively correlated with insulin resistance.^[39]^ Conversely, prooxidants promote oxidative stress by catalyzing radical production and causing cellular damage, such as excess iron in red meat.^[19,40]^ Cigarette smoke activates the NADPH oxidase-dependent ROS-sensitive AMP-activated protein kinase signaling pathway, inducing inflammation; it can also trigger secondary ROS release from inflammatory cells.^[41]^ There is a direct dose-dependent relationship between alcohol intake and blood pressure, with alcohol inducing oxidative stress by inhibiting antioxidant enzymes and causing inflammation.^[42]^ Obesity is often accompanied by chronic inflammation of adipose tissue, with pro-inflammatory signaling from adipocytes leading the immune system to release more pro-inflammatory factors and mediators, exacerbating oxidative stress.^[43,44]^

The OBS offers a composite, modifiable metric that may serve both as a stratification tool for risk assessment and a guide for non-pharmacological management in hypertensive patients. Current hypertension guidelines emphasize lifestyle modification as a cornerstone of treatment.^[45]^ A large population study suggests that controlling key risk factors such as blood pressure, BMI, and smoking can greatly reduce poor prognosis in patients with hypertension.^[46]^ Various dietary patterns have been shown to improve blood pressure and long-term prognosis in the prevention and treatment of hypertension.^[47–49]^ Our results suggest that adopting an antioxidant-rich diet and reducing prooxidant exposures could improve oxidative balance and ultimately prognosis. The OBS may thus aid clinicians in identifying high-risk individuals who could benefit from intensified dietary and behavioral interventions. Furthermore, as an accessible and integrative indicator, OBS could be incorporated into patient education and motivational frameworks to promote adherence to heart-healthy lifestyles.

However, several limitations should be considered. First, although we adjusted for extensive covariates, residual confounding may persist due to unmeasured factors such as physical activity, supplement use, and medication adherence. Second, the use of 24-hour dietary recall data from NHANES is subject to recall biases, which may affect the accuracy of nutrient intake estimates. Third, the possibility of reverse causality cannot be excluded; for instance, low OBS may reflect poor preexisting health status rather than directly influencing mortality. Finally, missing data for certain confounding factors were addressed via mean imputation, which may introduce minor bias; however, as these missing values accounted for <1% of observations, the results remain methodologically robust.

## 5. Conclusion

In patients with hypertension, there is a nonlinear relationship between OBS and SBP and all-cause mortality. Higher OBS is associated with reduced SBP levels and all-cause mortality risk in patients with hypertension, suggesting that OBS may be valuable in assessing and intervening in the long-term prognosis and mortality risk of patients with hypertension.

## Acknowledgments

We would like to thank Editage (www.editage.cn) and the DeepSeek AI tool for their assistance in English language editing.

## Author contributions

**Conceptualization:** Jie Li, Qiying Yao, Liang Chen.

**Data curation:** Jie Li, Liang Chen.

**Formal analysis:** Jie Li, Yue Guan, Chuang Sun, Jinzhou Zhu, Qiying Yao, Liang Chen.

**Funding acquisition:** Mengmeng Shan, Jinzhou Zhu.

**Investigation:** Yiming Zeng, Yue Guan, Chuang Sun, Mengmeng Shan, Qiying Yao.

**Methodology:** Jie Li, Yiming Zeng, Mengmeng Shan, Jinzhou Zhu, Liang Chen.

**Project administration:** Jinzhou Zhu, Qiying Yao, Liang Chen.

**Software:** Jie Li, Yiming Zeng, Jinzhou Zhu, Liang Chen.

**Supervision:** Jinzhou Zhu, Qiying Yao, Liang Chen.

**Validation:** Yiming Zeng, Yue Guan, Chuang Sun, Qiying Yao, Liang Chen.

**Visualization:** Jie Li, Yiming Zeng, Yue Guan, Chuang Sun, Jinzhou Zhu, Liang Chen.

**Writing – original draft:** Jie Li, Yiming Zeng.

**Writing – review & editing:** Yue Guan, Chuang Sun, Mengmeng Shan, Jinzhou Zhu, Qiying Yao, Liang Chen.

## Supplementary Material


