# Supplementary material for: The relationship between Oxidation Balance Score and all-cause mortality in patients with hypertension: An observational study
Source: Medicine (Baltimore). 2025 Nov 21;104(47):e46060. doi: 10.1097/MD.0000000000046060 (PMC12643772; doi:10.1097/MD.0000000000046060)

**Supplemental Table 1. Oxidative balance score assignment scheme**

| **OBS components** | **Property** | **OBS score** | | |
| --- | --- | --- | --- | --- |
|  |  | **0** | **1** | **2** |
| **Dietary Fiber (g/d)** | Antioxidative | ≤10.60 | 10.60-18.00 | ＞18.00 |
| **Beta-Carotene (mcg/d)** | Antioxidative | ≤326.00 | 326.00-1318.00 | ＞1345.00 |
| **Riboflavin (mg/d)** | Antioxidative | ≤1.39 | 1.39-2.17 | ＞2.17 |
| **Niacin (mg/d)** | Antioxidative | ≤16.43 | 16.43-25.79 | ＞25.79 |
| **Vitamin B_6_ (mg/d)** | Antioxidative | ≤1.27 | 1.27-2.06 | ＞2.06 |
| **Total Folate (mcg/d)** | Antioxidative | ≤253.58 | 253.58-414.86 | ＞414.86 |
| **Vitamin B_12_ (mcg/d)** | Antioxidative | ≤2.47 | 2.47-4.94 | ＞4.94 |
| **Vitamin C (mg/d)** | Antioxidative | ≤30.30 | 30.30-91.19 | ＞91.19 |
| **Vitamin E (mg/d)** | Antioxidative | ≤4.73 | 4.73-8.35 | ＞8.35 |
| **Calcium (mg/d)** | Antioxidative | ≤555.00 | 555.00-952.80 | ＞952.80 |
| **Magnesium (mg/d)** | Antioxidative | ≤206.85 | 206.85-292.00 | ＞292.00 |
| **Zinc (mg/d)** | Antioxidative | ≤7.26 | 7.26-11.67 | ＞11.67 |
| **Copper (mg/d)** | Antioxidative | ≤0.84 | 0.84-1.29 | ＞1.29 |
| **Selenium (mcg/d)** | Antioxidative | ≤75.97 | 75.97-118.71 | ＞118.71 |
| **Total fat (g/d)** | Pro-Oxidative | ≥86.88 | 53.17-86.88 | <53.17 |
| **Iron (mg/d)** | Pro-Oxidative | ≥15.61 | 9.87-15.61 | <9.87 |
| **Alcohol (g/d)** | Pro-Oxidative | ≥30 | 0-30 | 0 |
| **Cotinine (ng/mL)** | Pro-Oxidative | ≥0.208 | 0.022-0.208 | <0.022 |
| **Body Mass Index (kg/m^2^)** | Pro-Oxidative | ≥32.10 | 26.86-32.10 | <26.86 |

**Supplemental Table 2. Analysis of the correlation between OBS and blood pressure**

|  | **SBP** | **DBP** |
| --- | --- | --- |
|  | **β(95%CI)P** | **β(95%CI)P** |
| **OBS Continus** | -0.059 (-0.099, -0.018) 0.004 | -0.022 (-0.053, 0.009) 0.169 |
| **OBS Quartile** |  |  |
| Q1 | Ref. | Ref. |
| Q2 | 0.020 (-0.751, 0.791) 0.959 | -0.265 (-0.860, 0.330) 0.383 |
| Q3 | -0.096 (-0.901, 0.708) 0.814 | -0.885 (-1.505, -0.264) 0.005 |
| Q4 | -1.153 (-1.932, -0.375) 0.004 | -0.274 (-0.875, 0.326) 0.379 |
| P for trend | 0.002 | 0.233 |

Abbreviation: β, beta; CI, confidence interval; OBS oxidative balance score; SBP, systolic blood pressure; DBP, diastolic blood pressure.

Models adjusted for age, sex, race, education level, household income to poverty ratio, ALT, creatinine, uric acid, hyperlipidemia, diabetes, heart failure, coronary heart disease, and stroke.

**Supplemental Table 3. The effects per standard deviation increase in OBS on all cause mortality.**

|  | **Model 1** | **Model 2** | **Model 3** |
| --- | --- | --- | --- |
|  | **HR (95%CI) P** | **HR (95%CI) P** | **HR (95%CI) P** |
| **OBS for per standard deviation** | 0.89 (0.86, 0.92) <0.001 | 0.86 (0.83, 0.89) <0.001 | 0.94 (0.91, 0.98) 0.002 |
| **OBS for per standard deviation (Weighted)** | 0.86 (0.82, 0.90) <0.001 | 0.83 (0.79, 0.87) <0.001 | 0.75 (0.69, 0.83) <0.001 |

Model 1: No covariates adjusted.

Model 2: Adjusted age, sex, and race.

Model 3: Adjusted age, sex, race, education level, ratio of family income to poverty, ALT, creatinine, uric acid, hyperlipidemia, diabetes, heart failure, coronary heart disease, and stroke.

**Supplemental Fig 1. Smoothed curve fit between OBS and SBP all-cause mortality rates**


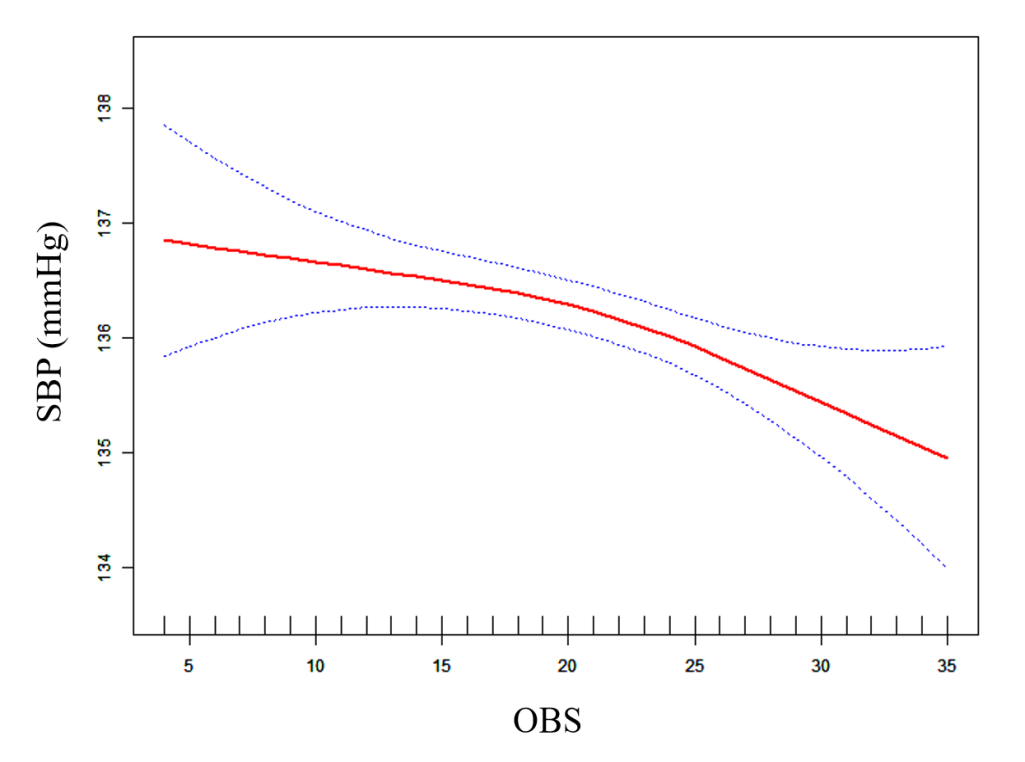


**Supplemental Fig 2. Smoothed curve fit between OBS and DBP all-cause mortality rates**


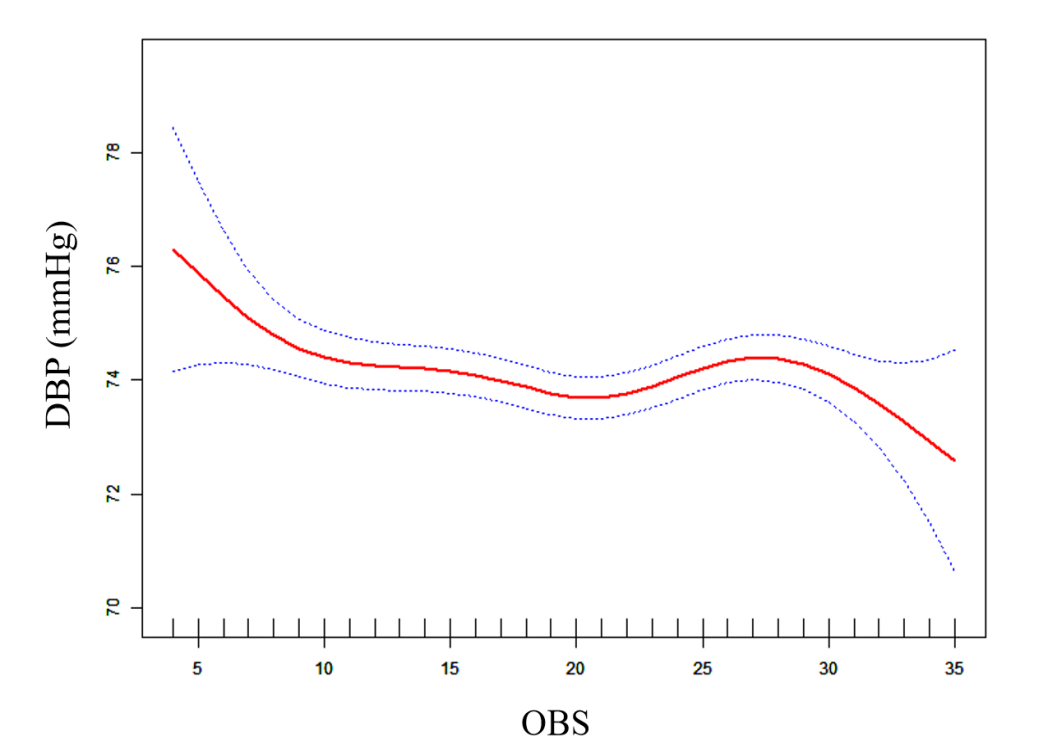

Supplement: Supplementary file 1 [file medi-104-e46060-s001.docx]
